# An Experimental Study on the Mechanical Properties and Microstructure of the Cemented Paste Backfill Made by Coal-Based Solid Wastes and Nanocomposite Fibers under Dry–Wet Cycling

**DOI:** 10.3390/ma17102256

**Published:** 2024-05-10

**Authors:** Haodong Wang, Qiangqiang Cheng, Nan Zhou, Heming Su, Qixiang Yin, Bin Du, Linglei Zhang, Yue Yao

**Affiliations:** 1 School of Mining Engineering, China University of Mining and Technology, Xuzhou 221000, China; ts22020055a31@cumt.edu.cn (H.W.); zhounanyou@126.com (N.Z.); 2 School of Architecture and Construction, Jiangsu Vocational Institute of Architectural Technology, Xuzhou 221000, China; yinqixiang1988@163.com (Q.Y.); dubin_china@163.com (B.D.); zlinglei@163.com (L.Z.); cm.522.1314@foxmail.com (Y.Y.); 3 China Shenhua Energy Co Ltd., Beijing 102209, China; 17810243324@163.com

**Keywords:** nanofibers, dry–wet cycle, mechanical properties, microscopic structure

## Abstract

The mechanical properties and microstructure of the cemented paste backfill (CPB) in dry–wet cycle environments are particularly critical in backfill mining. In this study, coal gangue, fly ash, cement, glass fiber, and nano-SiO_2_ were used to prepare CPB, and dry–wet cycle tests on CPB specimens with different curing ages were conducted. The compressive, tensile, and shear strength of CPB specimens with different curing ages under different dry–wet cycles were analyzed, and the microstructural damage of the specimens was observed by scanning electron microscopy (SEM). The results show that compared with the specimens without dry–wet cycles, the uniaxial compressive strength, tensile strength, and shear strength of the specimens with a curing age of 7 d after seven dry–wet cycles were the smallest, being reduced by 40.22%, 58.25%, and 66.8%, respectively. After seven dry–wet cycles, the compressive, tensile, and shear strength of the specimens with the curing age of 28 d decreased slightly. The SEM results show that with the increasing number of dry–wet cycles, the internal structure of the specimen becomes more and more loose and fragile, and the damage degree of the structural skeleton gradually increases, leading to the poor mechanical properties of CPB specimens. The number of cracks and pores on the specimen surface is relatively limited after a curing age of 28 d, while the occurrence of internal structural damage within the specimen remains insignificant. Therefore, the dry–wet cycle has an important influence on the both mechanical properties and microstructure of CPB. This study provides a reference for the treatment of coal-based solid waste and facilitates the understanding of the mechanical properties of backfill materials under dry–wet cycling conditions.

## 1. Introduction

Coal-based solid wastes refer to the mining waste generated during the mining process (such as coal gangue and fly ash) [1]. Coal-based solid wastes can cause several environmental issues. For instance, the open dumping of coal gangues results in the weak utilization of land resources, land subsidence, and the pollution of water resources [2]. To ensure the safe and efficient development of the coal industry, research on the disposal of generated solid wastes has been widely conducted. In the 1970s, several nations recognized the potential for the secondary utilization of waste resources and embarked on exploring the industrial value of coal gangue. Subsequently, these coal-based solid wastes have been applied for power generation, the preparation of ground engineering materials and chemical materials, agro-pharmaceutical preparation, and underground backfilling. Among them, power generation using coal gangue is the most prevalent method, where the washed coal gangue is transported from the mine to the power plant for electricity generation [3]. Additionally, coal gangue was mixed as a cementing material for road embankments or bridges in European countries [4]. The utilization rate of coal gangue in China is relatively high, and corresponding technical standards have been developed based on practical application methods.

At present, cemented paste backfill (CPB) made of coal-based solid wastes has been widely used in the mining field. In the preparation of CPB, solid wastes are used as aggregates, with a mass ratio of about 70–85%; additives with a mass ratio of about 3–7% are employed to improve the shear resistance of the materials [5]. The mechanical properties of cemented tailings backfill are extremely important for the safety of mineral resources and environmental improvement [6]. As an innovative cemented material for backfill mining, the cemented tailings backfill is transported to the goaf to support the overburden and alleviate the pressure in the working face. This approach also effectively reduces solid waste accumulation and mitigates associated environmental issues [7]. Additionally, solid waste plays a pivotal role in various domains. Incorporating rice husk ash into the production of ceramic tiles results in the firing of novel porous, lightweight, and high-temperature resistant ceramic tiles due to their silicate properties. This not only effectively addresses solid waste disposal for ecological preservation but also enhances the mechanical characteristics of materials utilized within the construction industry [8].

CPB can also provide support for mining roadways; prevent mining pressure disasters such as collapses, rib spalling, and roof caving; and effectively control surface subsidence [9]. For instance, when lithium slag and fly ash were used as composite additives, their application in cemented fine tailings backfill (CFTB) significantly improved, fully meeting the support requirements of the surrounding rock in the mine [10]. The mechanical properties of the CPB are the main influencing factor for the stability of the working face during backfill mining [11]. Generally, CPB is mostly composed of tailings, cement, and other mixed solids [12]. However, the mechanical properties of backfill materials are limited and fail to fully withstand the high stress and complex disturbance environment encountered in mining faces. Consequently, the supporting function of the CPB body is compromised [13].

Nanocomposite fibers, as novel types of additive materials, have a positive impact on the enhancement of backfill materials. Nanocomposite fibers are types of crystalline materials that are susceptible to chemical reactions with other materials [14]. These fibers possess numerous advantages, such as their high mechanical strength and reduced environmental pollution risk [15,16]. When different mass ratios of nanocomposite fiber materials are added, the mechanical properties of the cemented material (such as the elastic modulus) are notably improved [17,18]. Existing research has found that a small amount of nanocarbon fibers have a considerable impact on the performance of composite materials. When 0.3% nanocarbon fibers is added, the mechanical properties of the modified materials are largely improved, and its shear strength increases by 17.5% [19]. During backfill mining, nanocomposite fiber–CPB with improved mechanical properties can fully alleviate the migration of the overburden and effectively control the deformation of roadway-surrounding rock. For example, carbon nanotubes (CNTs) can be used to alter the damage degree of the cement matrix and increase the chemical energy of the material, leading to a significant improvement in the compressive strength of the cement matrix [20]. In addition, polypropylene fibers can be used to modify rubber tailings and waste rock fillers [21]. Increasing the fiber length can improve the crack resistance of fiber-reinforced cementitious tailings waste rock fill. This newly formulated cemented backfill material fully satisfies the support strength requirements for backfill mining.

Research on the mechanical properties and damage laws of rock masses under dry–wet cycles has been extensively carried out [22]. P. A. Hal and A. Shakoor [23] investigated the development of irreversible damage to sandstone under dry–wet cycles. M. Duda and J. Renner [24] found that water can weaken the mechanical properties of sandstone. Yao [25] conducted triaxial compression tests on red sandstone after dry–wet cycles and explored the changing laws in the mechanical indicators of rock mass after dry–wet cycles. Wang [26] analyzed the effect of different pH values in the water environment on the erosion of muddy sandstone after dry–wet cycles. Through dry–wet cycle tests, Esfandiari. Z [27] examined the creep properties of two unsaturated sand–bentonite mixes and summarized the associated creep law. Zhang [28] conducted shear creep tests on granite specimens subjected to dry–wet cycling and determined the performance degradation and changes in creep properties under hydraulic weathering conditions. Guo [29] studied the impact of wet and dry cycles on specimens under sulfate solution erosion and analyzed the microstructure of concrete before and after erosion using scanning electron microscopy (SEM). In addition, the mechanical properties and mechanisms of the backfill materials under the dry–wet cycles is the key for failure analysis. Huang Kang et al. analyzed the influence of different loading angles on the fracture toughness of red mudstone materials after dry–wet cycles and studied the number and distribution of cracks in the fracture process of materials after different cycles [30]. Xin Cai et al. conducted research on the mechanical properties of sandstone; tested the longitudinal wave velocity of sandstone under different dry–wet cycles; and observed the crack state, intergrain cementation degree, and pore size of sandstone under different cycles from a microscopic perspective [31]. The above studies contribute to the understanding of the damage mechanism of sandstone after dry–wet cycles.

The current study primarily focuses on the impact of dry–wet cycles on rock properties within geotechnical engineering projects, such as surface reservoirs, dams, and highways, with limited exploration in the field of backfill materials. For modified CPB, their mechanical properties and the degree of microstructural damage under dry–wet cycles require further research. In this study, the macroscopic mechanical properties and microscopic damage degree of nanocomposite fiber–CPB under dry–wet cycles were analyzed. The effects of different dry–wet cycles and curing ages on the mechanical properties of nanocomposite fiber–CPB were analyzed, and a reduction in the mechanical properties and the microscopic damage of the specimens after the dry–wet cycle were discussed. This study facilitates the assessment of the damage degree of CPB in dry–wet cycle environments and effectively addresses the issue of solid waste disposal in coal mining.

## 2. Experimental Contents and Methods

### 2.1. Test Materials and Equipment

#### 2.1.1. Test Materials

**Figure 1:**
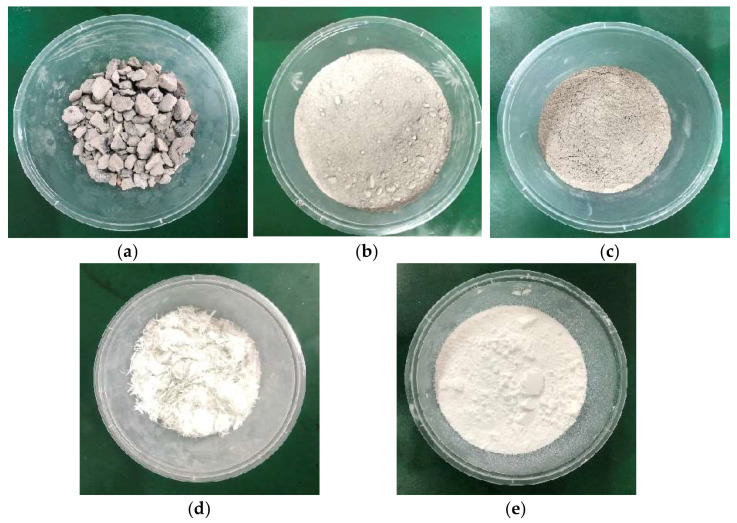
Materials required for preparation: (**a**) coal gangue; (**b**) fly ash; (**c**) cement; (**d**) glass fiber; (**e**) nano-SiO_2_.

The materials used in this study mainly included coal gangue, fly ash, cement, glass fiber, nano-SiO_2_, and water, as shown in Figure 1.

The coal gangue used in the test was taken from a mine of Shenhua Group in Inner Mongolia, China. The coal gangue was irregular in shape and exhibited a bright gray color. A jaw crusher was employed to crush the gangue to less than 20 mm. Fly ash was commercially purchased, and grade III fly ash with a grayish–white color was used. It was predominantly in powder form, with minimal clumping observed. PO42.5 Portland cement, glass fiber with a specification of 6 mm, and spherical nano-SiO_2_ with an average particle size of 20 nm were selected for the preparation of specimens [30,31]. Table 1 shows the performance parameters of the raw materials.

#### 2.1.2. Test Equipment

**Figure 2:**
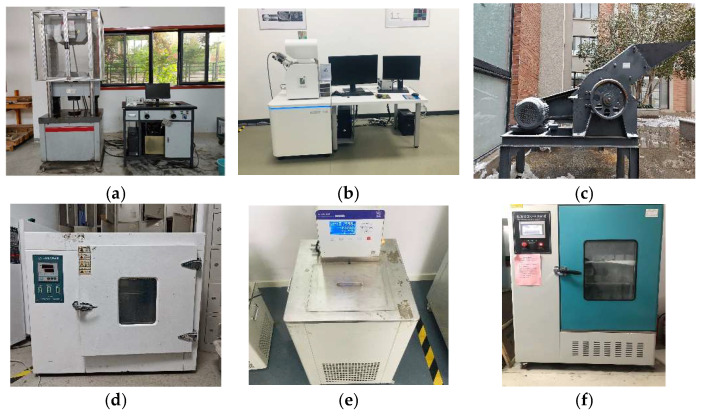
The equipment photographed is as follows: (**a**) press; (**b**) SEM; (**c**) jaw crusher; (**d**) drying oven; (**e**) thermostatic water tank; (**f**) standard curing box.

To perform the mechanical characterization and microstructure testing, a servo press, a jaw crusher, a scanning electron microscope, a constant temperature water tank, a drying oven, a standard curing box, and a handheld mixer were used in this study. Figure 2 shows certain photos of the equipment, and Table 2 lists the specific parameters of the equipment and manufacturer information.

### 2.2. Testing Schemes

Based on engineering practice and research continuity [30,31,32], the solid mass ratio of nanocomposite fiber–CPB was set as 79% in this test. Table 3 shows the mass share of each material, where the mass ratio indicates the ratio of the total mass of the item to the material. Existing studies have found that cement-based materials with a 1% mass fraction of nanoscale additives exhibit better mechanical properties [32,33,34]. Therefore, a 1% mass fraction of nano-SiO_2_ was added in this test. It should be noted that due to the relatively small mass fractions of glass fiber and nano-SiO_2_, their mass ratios can be ignored.

According to the above ratios, the prepared specimens were prepared and subjected to curing ages of 7 d, 14 d, and 28 d (curing age indicates the time for which the specimen was placed in the standard curing box), respectively. Then, the wet–dry cycle test was conducted on the specimens after curing. Specifically, the dry–wet cycle test was carried out using the ASTMD4843-88 [35]. Each cured specimen was placed in a drying oven at a temperature of 60 °C and dried for 24 h; then, each specimen was immersed in a constant temperature water tank at 20 °C for 24 h [36]. The entire procedure was considered as a single dry–wet cycle. The total number of cycles was set to 0, 1, 3, and 7, respectively. Finally, mechanical tests and microstructure characteristic tests on these specimens were conducted under different curing ages and dry–wet cycles. Table 4 shows the details of the test plan.

### 2.3. Specimen Preparation

**Figure 3:**
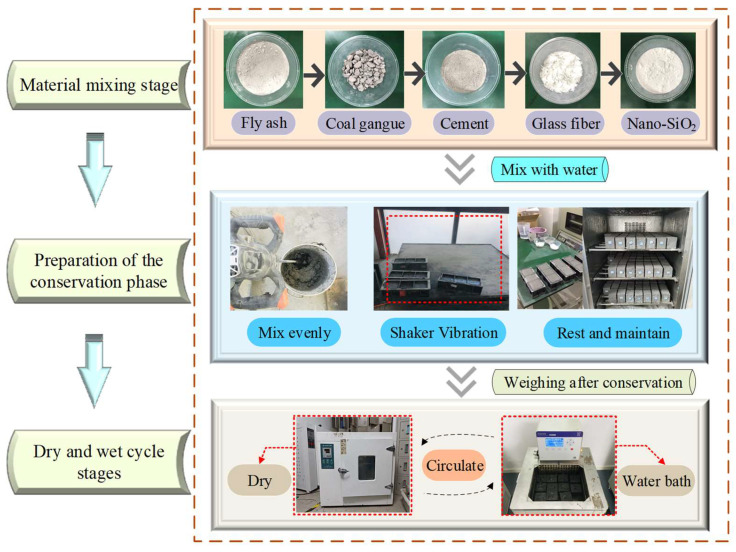
Specimen preparation process.

Three specimens were prepared in each group, and the average value of the three specimens after experiments were taken as the final result. The fly ash, coal gangue, cement, and glass fiber were added to the mixing bucket for dry mixing. After the ingredients were evenly mixed and fully in contact, nano-SiO_2_ was added for dry mixing. Subsequently, tap water was slowly added for stirring, and the mixture was stirred for 4–5 min. The prepared slurry, at a specified ratio, was poured into a standard mold [37]. A small amount of engine oil was applied evenly on the sides and bottom of the mold, and the vibration table was activated to achieve uniform vibration when the mold volume was filled with the prepared slurry. After the entire mold was filled, the slurry was stranded for 24 h, the mold was removed, and the specimen was numbered and placed in a curing box at a temperature of 20 ± 0.5 °C and a humidity of 95% RH [38]. After that, the prepared specimen was subjected to dry–wet cycles. Figure 3 shows the specific process followed for the preparation of the specimens.

## 3. Results and Discussion

### 3.1. Compressive Strength Test

#### 3.1.1. The Effect of Different Dry–Wet Cycles on Compressive Strength

**Figure 4:**
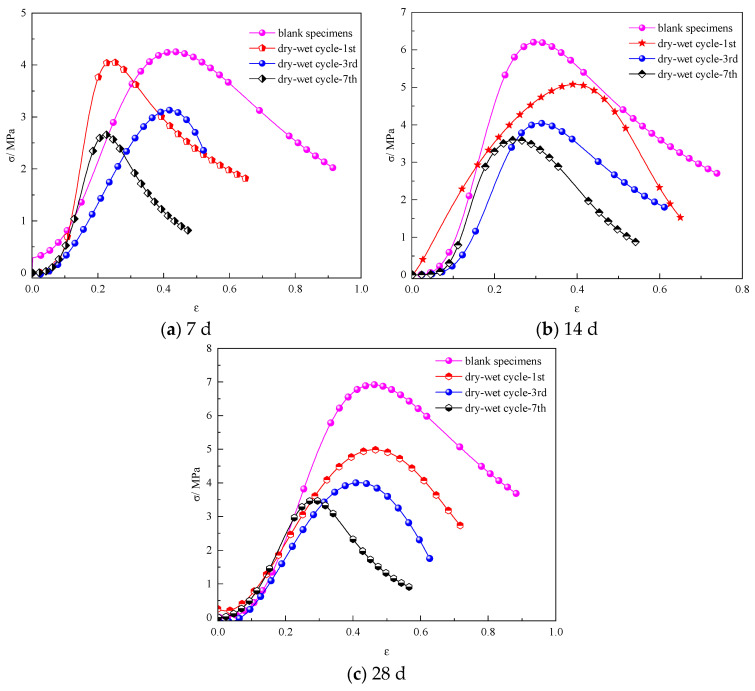
Stress–strain curves of the compressive strength of the specimens at different curing ages after different wet–dry cycles: (**a**) 7 d; (**b**) 14 d; (**c**) 28 d.

The stress–strain curves of the compressive strength of the specimens were obtained after different numbers of wet–dry cycles, as shown in Figure 4.

Regarding the early curing stage, the decrease in the compressive strength of specimens after one to three dry–wet cycles is insignificant, while the compressive strength of the specimens decreased notably after four to seven dry–wet cycles. However, the peak stress of the specimens decreased notably with an increase in the number of dry–wet cycles. As the curing age increases, the compressive strength of the specimens increases considerably. After 28 d of curing, the peak stress of the specimens free from dry–wet cycling was 7.25 MPa, and with the increasing number of dry–wet cycles, the peak stress of the specimens decreased continuously; the peak stress of the specimens after three and seven dry–wet cycles decreased to 4.1 Mpa and 3.5 Mpa. Although the peak stress values of the specimens varied under different cycles, the stress curves tended to be flat, and a certain residual strength was maintained, indicating that the cemented material still has a certain toughness after the dry–wet cycle [39].

Therefore, increasing the number of dry–wet cycles results in a reduction in the compressive strength of the specimens in the early stage. However, after a certain number of dry–wet cycles, the decrease in the peak stress of the specimens slows down in the later stage.

**Figure 5:**
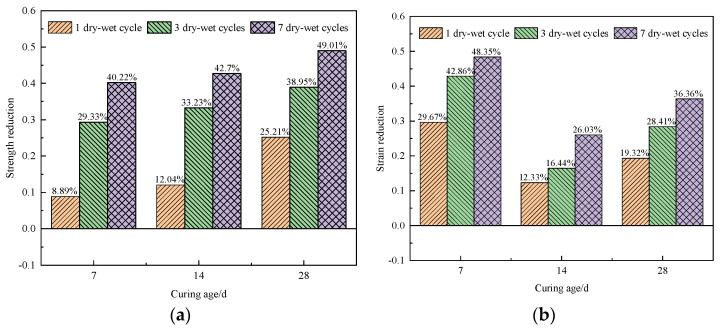
Effect of the number of dry–wet cycles on the stress–strain reductions the of specimens: (**a**) stress increase; (**b**) strain increase.

The degree of the stress and strain reductions of the specimen after one to three dry–wet cycles was obtained, as shown in Figure 5.

After 7 d of curing, the peak stress of the specimens after one, three, and seven dry–wet cycles decreased by 8.89%, 29.33%, and 40.22%, respectively. Compared to the specimens free from the dry–wet cycling, the peak stress of the specimens after seven dry–wet cycles decreased by 40.22%. With an increase in the number of dry–wet cycles, the peak compressive strength of the specimens decreased, indicating that the compressive strength of cemented backfill materials gradually deteriorates due to the dry–wet cycles [40]. When cured for 28 d, the compressive strengths of the specimens after one and three dry–wet cycles decreased notably. Compared to the specimens without a dry–wet cycle, the peak stress of the specimen with one dry–wet cycle decreased by 25.21%. However, as the number of dry–wet cycles increased from one to seven, the decreasing rate of the stress slowed down. This may be because the damage degree caused by the dry–wet cycle inside the specimens tends to be saturated, resulting in a continuous decrease in the reduction rate regarding the compressive strength of the specimens.

Under conditions of dry–wet cycling, the strain corresponding to the peak stress of the specimens decreased. After seven dry–wet cycles, the maximum decreases in the strain of the specimens after curing ages of 7d, 14d, and 28d were 48.35%, 26.03%, and 36.36%, respectively. The results show that dry–wet cycling has a negative effect on the strain of the specimens and that the curing age also affects the strain reduction in the specimens; during the dry–wet cycling, the early strain exhibited a greater reduction compared to the later strain, while no significant linear correlation was observed between the specimens’ strain values and the number of dry–wet cycles.

#### 3.1.2. Effect of Curing Age on Compressive Strength of Specimens after Dry–Wet Cycles

**Figure 6:**
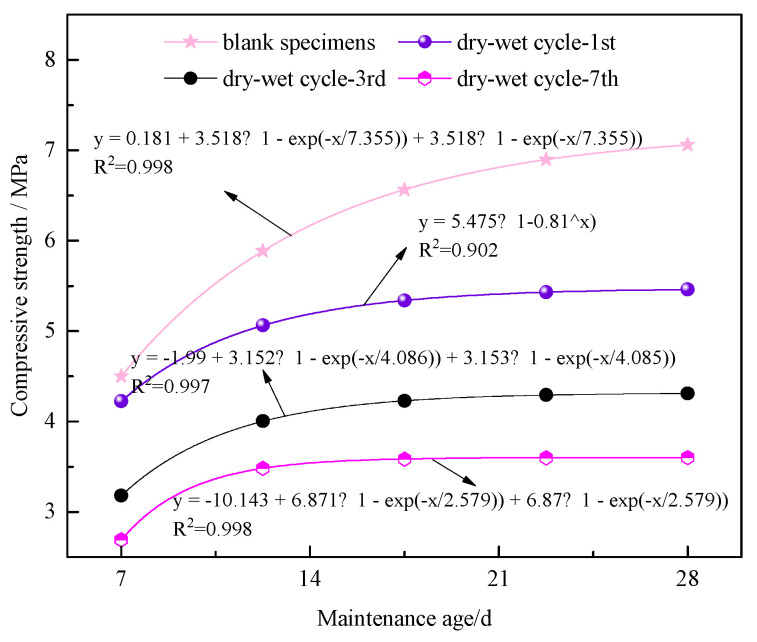
Relationship between curing age and compressive strength of specimens.

As shown in Figure 6, the peak compressive strengths of the specimens at different curing ages and after varying cycles were analyzed.

Compared with the blank control specimens, the mechanical properties of the nanocomposite fiber–CPB specimens after dry–wet cycling decreased notably, indicating that the compressive strength of the specimens is inhibited to a certain extent after dry–wet cycles. With an increase in the curing age, the slope of the fitted curve of peak compressive strength is higher before the curing age of 14 d. The increasing trend of peak stress in the later curing stage (after 14 d curing) is not significant, and the dry–wet cycling has a greater influence on the compressive resistance of the specimen before 14 d curing. According to the fitting equations of each cycle number, the fitting degree R^2^ of the blank specimens and tested specimens after three and seven dry–wet cycles is above 0.997. It can be found that with an increase in the curing age, the increasing amplitude of the peak stress of the specimen gradually decreases and eventually tends to be flat, which also indicates that under different dry–wet cycles, the compressive strength of the specimen has a negative exponential increasing relationship with the curing period (x ≤ 28) [41].

**Figure 7:**
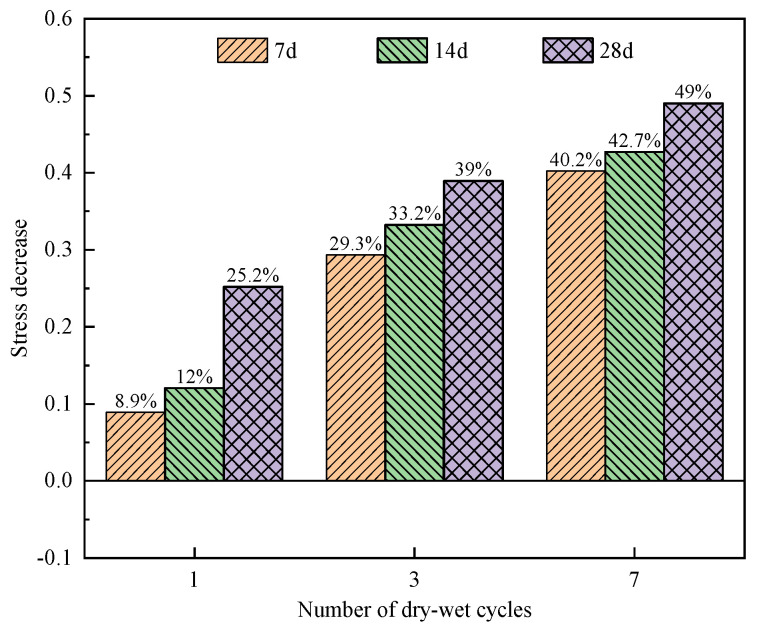
Reductions in specimen stress at different curing ages.

Figure 7 illustrates the stress reductions for the specimens after different curing ages.

Compared to the specimens with a curing age of 7 d and without a dry–wet cycle, the stress in the specimens with a curing age of 7 d after one and three dry–wet cycles decreased by 8.9% and 29.3%, the stress of the specimens after seven dry–wet cycle decreased by 40.2%, and the decreasing rate slowed down notably. After 28 d of curing, compared to blank specimens, the peak stress of the specimens after different dry–wet cycles decreased notably. As shown in Figure 7, with the increasing number of dry–wet cycles, the decreasing rate of stress gradually slows down. This is because the degree of damage to the specimen caused by the dry–wet cycles tends to be saturated, and the reduction rate regarding the peak stress of the specimen decreases continuously.

### 3.2. Tensile Strength Test

#### 3.2.1. The Effect of Different Dry–Wet Cycles on Tensile Strength

**Figure 8:**
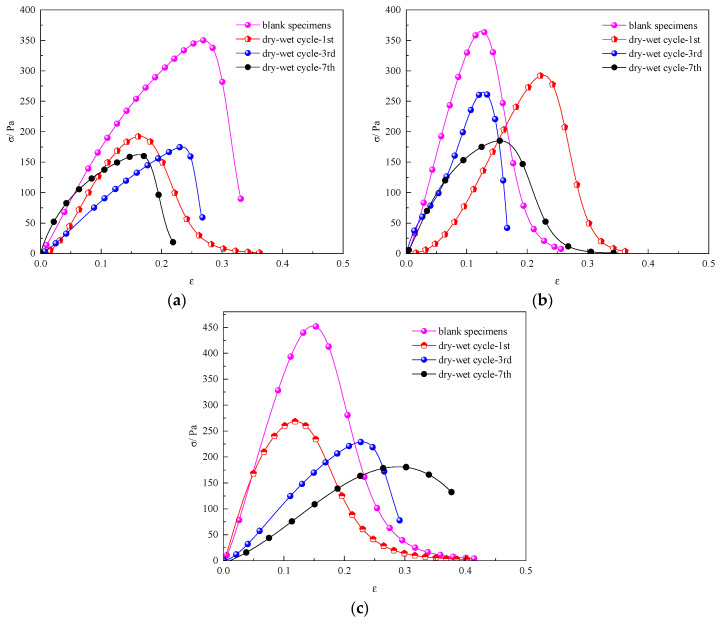
Tensile strength of specimens at varying curing ages after different dry–wet cycles: (**a**) 7 d; (**b**) 14 d; (**c**) 28 d.

Figure 8 illustrates the tensile strength of the nanocomposite fiber–CPB specimens after different dry–wet cycles.

As illustrated in Figure 8, with an increase in the number of dry–wet cycles from one to seven, the stress of the specimens at different curing ages decreases to varying degrees. When the curing ages were 7 d, 14 d, and 28 d, a considerable decrease in the peak stress of the specimens occurred after one dry–wet cycle. Furthermore, the maximum reduction in the peak stress of the specimens after seven dry–wet cycles can be observed.

**Figure 9:**
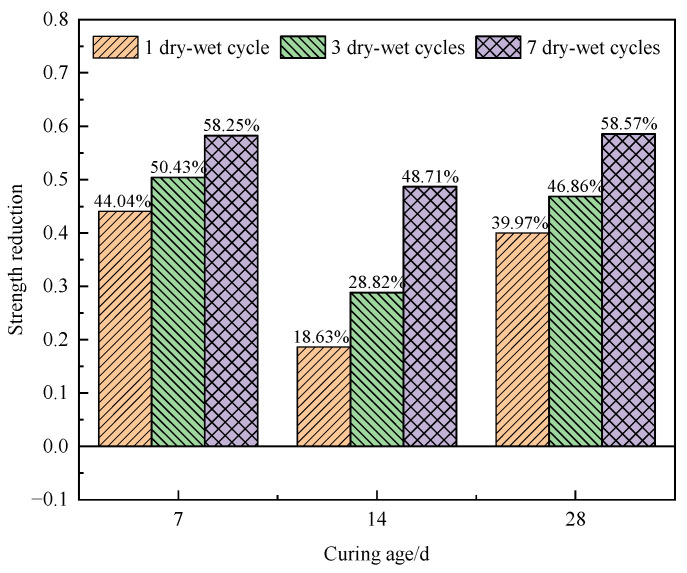
Reductions in tensile strengths of specimens under different dry–wet cycles.

Figure 9 shows the reductions in the tensile strengths of the specimens under different dry–wet cycles compared to those without dry–wet cycles.

As shown in Figure 9, at curing ages of 7 d, 14 d, and 28 d, compared to the specimens without dry–wet cycling, the stress of the specimens subjected to dry–wet cycling decreased to varying degrees, and the decrease in stress gradually increased with an increase in the number of dry–wet cycles from 1 to 7. At curing ages of 7 d or 28 d, the tensile strength of the specimens decreased notably after seven dry–wet cycles, with a maximum reduction of about 58%. It can be found that when the curing age is less than 7 d or more than 28 d, the influence of the dry–wet cycling on the specimen is worse.

#### 3.2.2. Effect of Curing Age on Tensile Strength of Specimens after Dry–Wet Cycles

**Figure 10:**
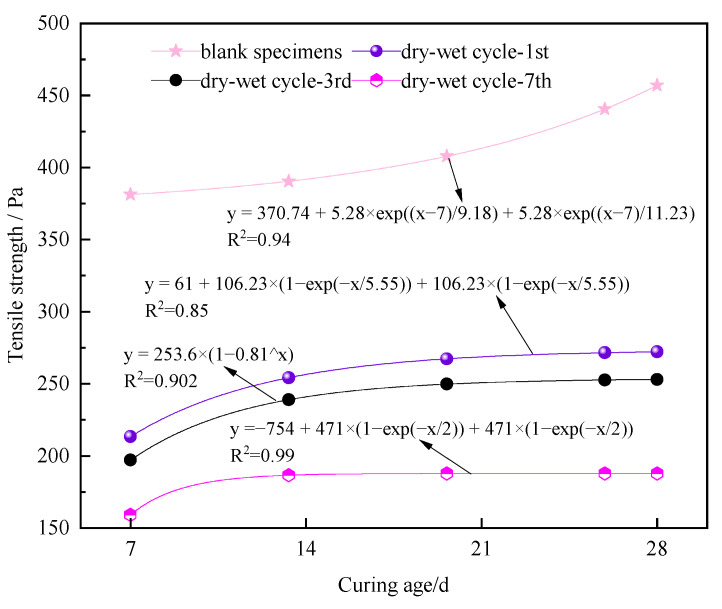
Relationship between curing age and tensile strength of specimens.

As shown in Figure 10, the peak tensile strengths of the specimen at different curing ages after zero, one, three, and seven dry–wet cycles were analyzed by curve fitting.

The tensile strength and curing age of the specimens without dry–wet cycles, as well as those subjected to three and seven dry–wet cycles, exhibit a linear correlation. The fitting degree R^2^ of the specimens is above 0.902. The tensile strength of the specimens subjected to one, three, and seven dry–wet cycles shows a decreasing trend with the increase in curing age. However, for when the curing age was 7 d, the slope of the fitted curve of tensile strength decreases, and the curve tends to flatten.

### 3.3. Shear Strength Tests

#### 3.3.1. The Effect of Different Dry–Wet Cycles on Shear Strength

**Figure 11:**
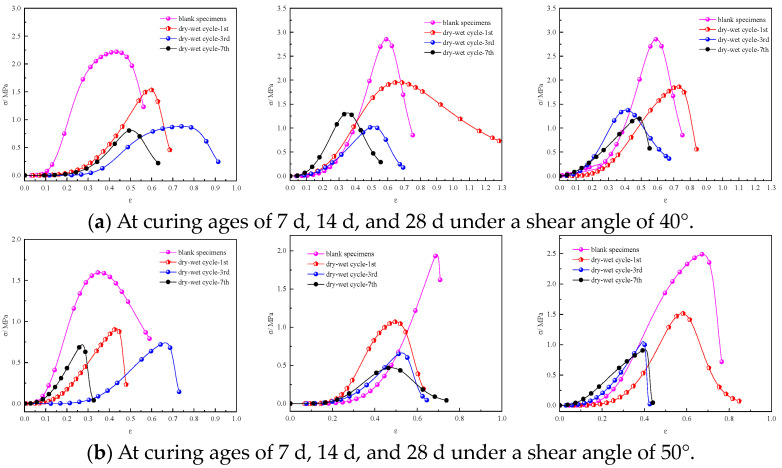

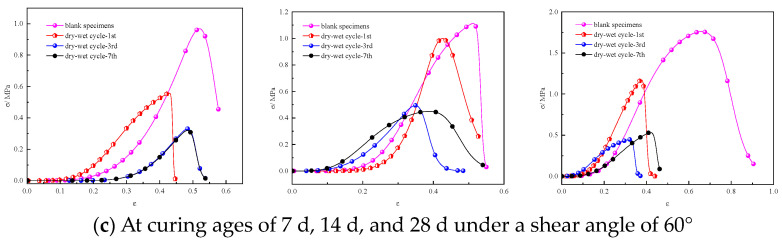
Shear strengths of specimens at curing ages of 7 d, 14 d, and 28 d after dry–wet cycling at different shear angles: (**a**) 40°; (**b**) 50°; (**c**) 60°.

Figure 11 shows stress–strain curves for the shear strengths of the specimens after varying dry–wet cycles at different shear angles. 

When the shear angle was 40°, the shear strength of the specimens after the dry–wet cycles was lower than that of the specimens without dry–wet cycling. As the number of dry–wet cycles increased from 1 to 7, the shear strength of the specimens decreased. When the shear angle was 50°, the shear strength decreased with an increase in the number of dry–wet cycles from one to seven. Under different curing ages, the shear strength of the specimens after one dry–wet cycle decreased notably. When the curing age was 14 d, the shear strength of the specimens after one dry–wet cycle exhibited the fastest decrease. When the shear angle was 60°, there was a relatively small reduction in the shear strength of the specimens with a curing age of 14 d after one dry–wet cycle. When the curing age was 28 d, the shear strength of the specimens decreased with the increasing number of dry–wet cycles.

**Figure 12:**
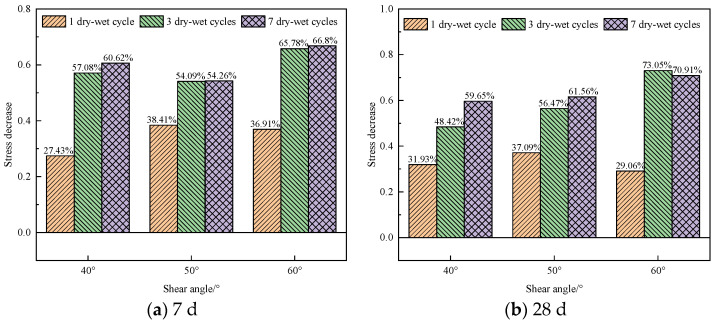
Effect of dry–wet cycling on shear strength of specimens at different curing ages: (**a**) 7 d; (**b**) 28 d.

Figure 12 shows the influence of the dry–wet cycling on the shear strength of the specimens in the early and late curing periods. At curing ages of 7 d and 28 d, the early shear strength of the specimens decreased notably. Compared with the blank specimens, the shear strength of the specimens after one, three, and seven dry–wet cycles at a shear angle of 40° decreased by 27%, 57%, and 61%, respectively. For when the shear angle was 50° or 60°, the trend of the shear strength reduction is similar to that at a shear angle of 40°. However, after three dry–wet cycles, the reduction in the shear strength of the specimens rarely changed. The maximum reduction in shear strength can be observed for specimens at a curing age of 7 d after seven dry–wet cycles, and the stress decreased by 61%, 54%, and 67% at shear angles of 40°, 50°, and 60°, respectively. Therefore, multiple dry–wet cycles can reduce the shear strength of the specimens with a short curing age, but the difference in the reduction amplitude between different shear angles is not significant.

In the later curing stage, compared with the specimens without dry–wet cycling, the shear strength of the specimens after the dry–wet cycling decreased by more than 29%. When the curing age was 28 d, the peak stress of the specimens with a shear angle of 60° decreased the most, decreasing by more than 73%. The dry–wet cycling has a considerable impact on the later shear strength of the specimen, and the reduction in shear strength exhibits a progressively greater magnitude as the number of dry–wet cycles increases. 

#### 3.3.2. Effect of Curing Age on Shear Strength of Specimens after Dry–Wet Cycle

Table 5 shows the shear strengths of the specimens at different angles under different curing ages. 

**Figure 13:**
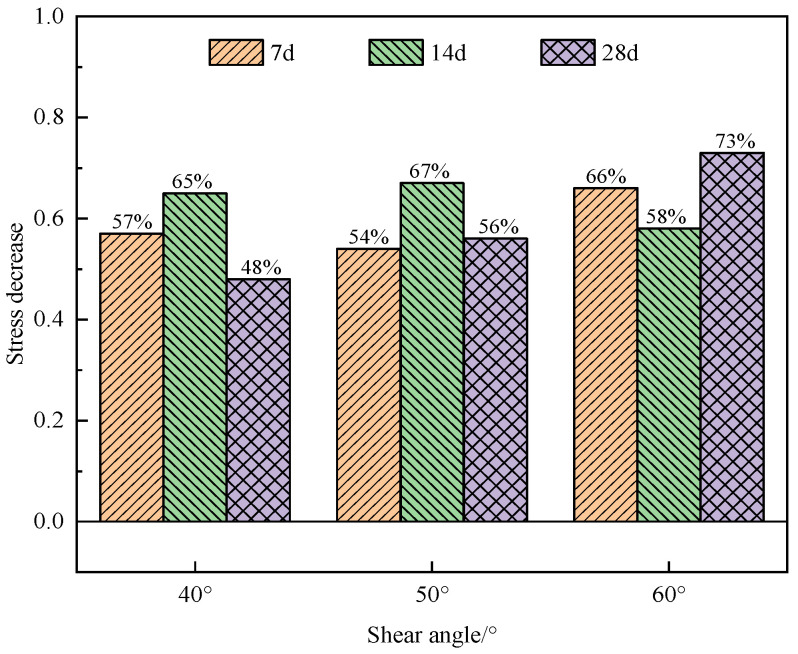
Plot of stress reductions for specimens at different curing ages after 3 dry–wet cycles.

Figure 13 shows the reductions in the shear strengths of the specimens after different curing ages under three dry–wet cycles.

As shown in Figure 13, after three dry–wet cycles, the shear strength of the specimens decreased notably at different shear angles. When the shear angle was 40°, the shear strength of the specimens increased with the increase in curing age. At a curing age of 14 d, the shear strength of the specimens exhibited a maximum reduction of 65% compared to the specimens without dry–wet cycling. When the shear angle was 50° and the curing age was 14 d, the maximum stress reduction of the specimens was 67%. When the shear angle was 60° and the curing age was 28 d, the stress reduction reached 73%.

Based on the analysis of the above results, it can be seen that the shear strength of the specimens decreases with an increase in the shear angle and that it increases with increasing curing age. When the shear angle is plotted on the *x*-axis and the curing age is plotted on the *y*-axis, different surface fitting functions can be used for fitting, and the shear strength surface equations are obtained as follows:

(1) $$f_{\left(x,y\right)}=0.98+0.026x+4{.76\;\times \;10}^{-4}y-\;6{.33\;\times \;10}^{-4}x^{2}-\;4{.54\;\times \;10}^{-4}y^{2}$$

(2) $$f_{\left(x,y\right)}=2.41\;-\;0.037x+0.0168y$$

(3) $$f_{\left(x,y\right)}=0.25+0.04058x+0.04512y-\;6{.33\;\times \;10}^{-4}y^{2}-\;8{.93\;\times \;10}^{-4}\mathrm{xy}$$

**Figure 14:**
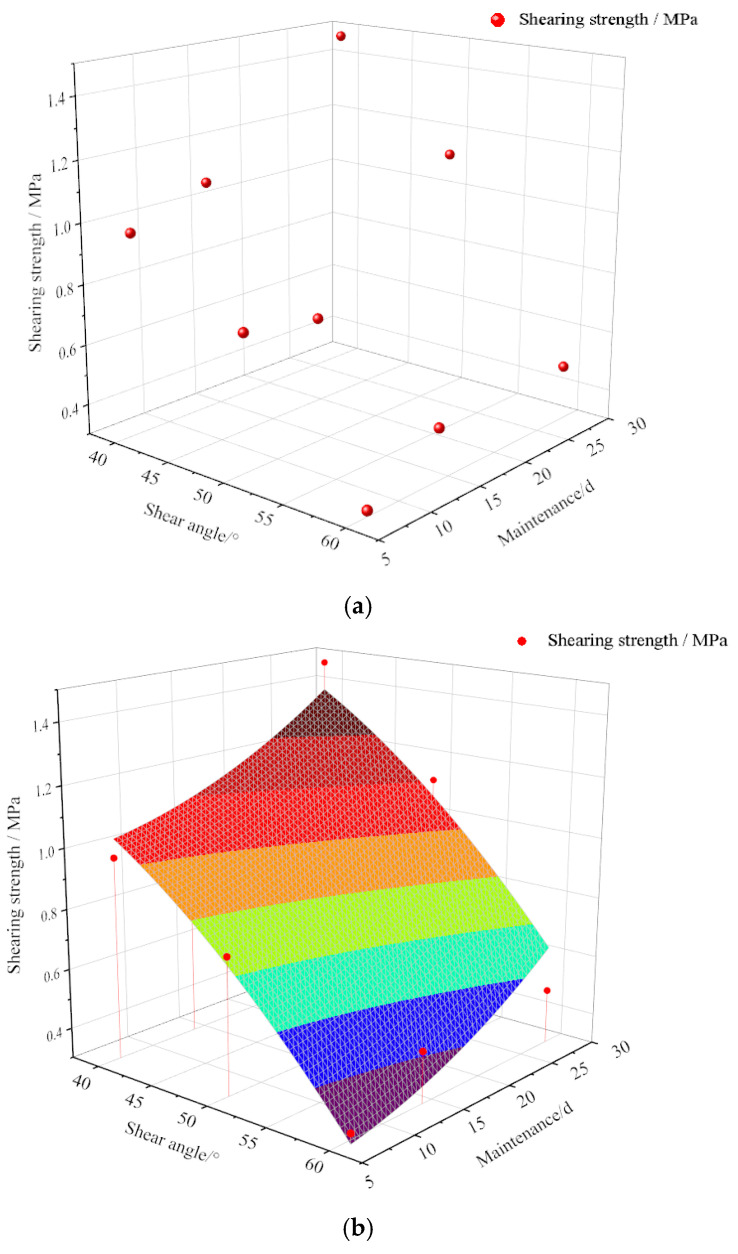

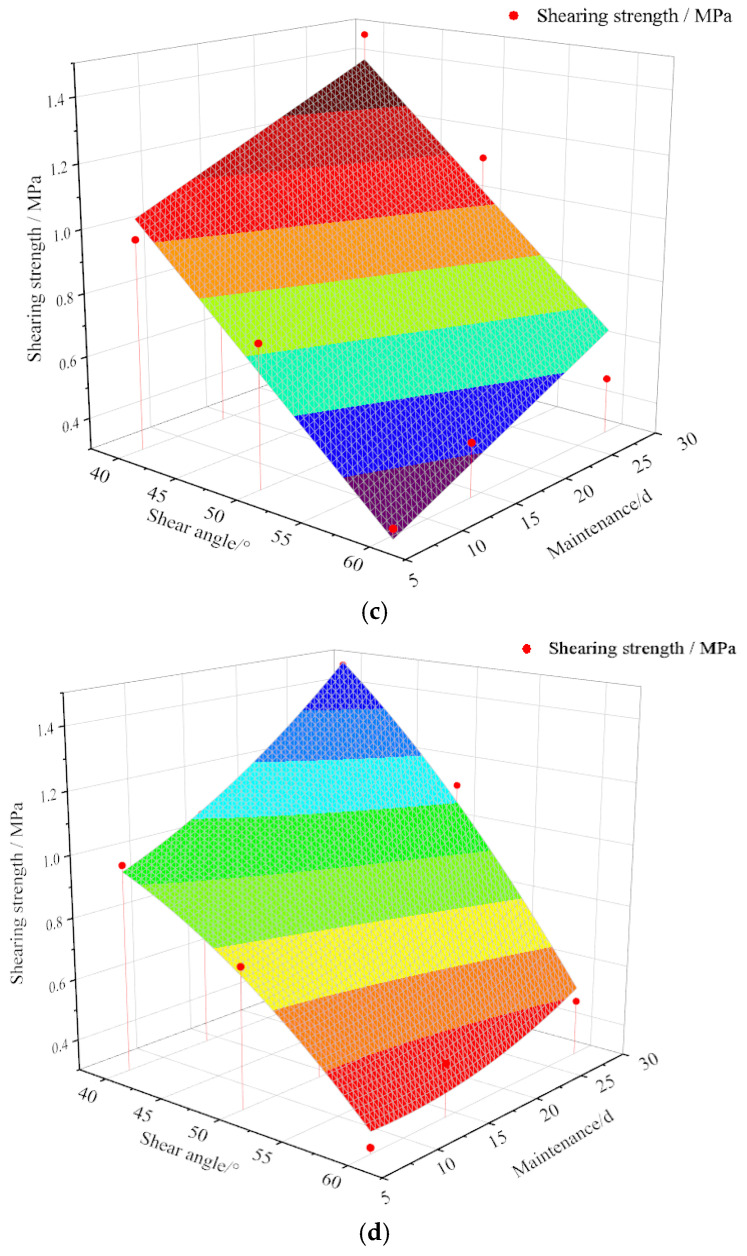
Relationship between curing age and shear strength of specimens: (**a**) shear strength Point plot; (**b**) Function 1: shear strength fitted surface; (**c**) Function 2: shear strength fitted surface; (**d**) Function 3: shear strength fitted surface.

Figure 14 shows the surfaces fitted with different functions. The sum of the squared residuals of each surface equation is 0.8898, 0.9233, and 0.9779, respectively. Equation (3) demonstrates the optimal fitting degree for the shear strength of the nanocomposite fiber–CPB specimen, and the R^2^ reaches an impressive value of 97.8%.

### 3.4. Analysis of Microstructure

To better analyze the mechanical damage of the specimen under different influencing factors, a VEGAGM electron microscope scanning instrument was selected to analyze the internal damage of the specimens.

#### 3.4.1. Microscopic Structure of Specimens after Different Dry–Wet Cycles

**Figure 15:**
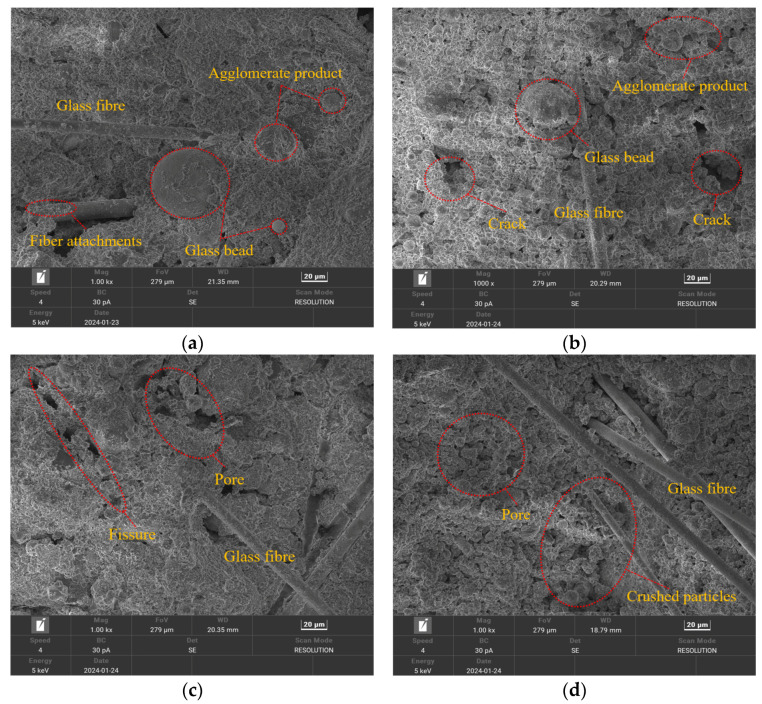
Microscopic structures of specimens after different dry–wet cycles: (**a**) free from the dry–wet cycle; (**b**) 1 dry–wet cycle; (**c**) 3 dry–wet cycles; (**d**) 7 dry–wet cycles.

In this section, a standard specimen with a curing age of 28 d is taken as an example; the microstructures of the specimens under different dry–wet cycles were observed, as shown in Figure 15.

As shown in Figure 15a–d, the microstructure of the cemented material undergoes considerable changes under different dry–wet cycles. The material at a curing age of 28 d undergoes a hydration reaction, and glass microspheres and agglomerates are generated. The two hydration products adhere to each other on the surface of the specimen, and there are no obvious cracks or pores on the surface of the specimen. As the number of dry–wet cycles increases, the surface morphology of the material undergoes considerable changes after being soaked in water. These changes are mainly reflected in the size of particles, the distribution of the internal structure, and the morphology of particles. When the specimen is not subjected to a dry–wet cycle, its microstructure is relatively dense and uniform, and the particle contours of the material are clear, with similar sizes and morphologies. When starting the dry–wet cycle, the particles are arranged relatively neatly and densely, with a small number of small pores, and the overall structure is relatively complete. After three dry–wet cycles, the gaps between the particles of the material gradually expand, and the internal clustered products gradually rupture, making the structure more loose and fragile [42]. Consequently, a porous and loose microstructure is gradually formed. After seven dry–wet cycles, the degree of deterioration gradually deepens, the size of the pores increases, and certain cracks are generated. The clustered products almost disappear, and the internal structural framework is damaged to some extent. The surface of the specimen becomes fragmented and easily peeled off, showing poor mechanical properties.

#### 3.4.2. Microscopic Structure of Specimens at Different Curing Ages

**Figure 16:**
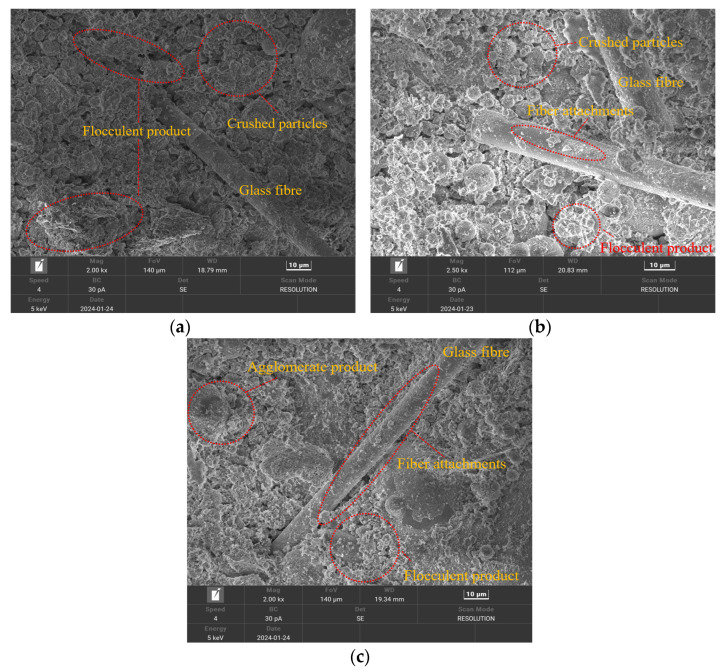
Microscopic structures of specimens at different curing ages: (**a**) 7 d; (**b**) 14 d; (**c**) 28 d.

In this section, a standard specimen after seven dry–wet cycles is taken as an example; the microstructures of specimens under different curing ages are shown in Figure 16.

As shown in Figure 16a–c, the microstructure of the cemented material undergoes different changes at different curing ages. After the hydration reaction, glass microspheres, agglomerates, flocculent products, and other products are generated. As the curing age increases, the hydration products prominently attach to the surface of the specimen, and there are relatively fewer cracks and pores on the surface of the specimen, resulting in a low degree of structural damage within the specimen. Regarding the specimen with a curing age of 7 d, its microstructure is relatively loose, and the particle contour of the specimen is fuzzy, with different particle sizes and a considerable degree of damage. Regarding the specimen with a curing age of 14 d, the particles are arranged relatively neatly and densely, a small number of products are attached to the surface of the glass fiber, overlapping with each other, and the specimen is more compacted. There are also a few small pores inside the specimen, and the overall structure is relatively complete. For the specimen with a curing age of 28 d, clusters of flocculent substances are generated, and the number of particles notably increases. The adhesion of flocculent substances on the fiber surface causes them to bond to each other [43]. This indicates that the friction between the various products of the specimen is enhanced and that the mechanical properties are improved. 

## 4. Conclusions

In this study, the mechanical properties and microstructure of composite nanofiber-cemented backfill materials were studied. Compressive, tensile, and shear tests were carried out using an electro-hydraulic servo press, and the effects of different dry–wet cycles and curing ages on the mechanical properties of the specimens were analyzed. In addition, SEM equipment was used to ascertain the development of cracks in the specimens after the dry–wet cycles [44]. The conclusions are as follows:

(1) With the increase in the number of dry–wet cycles, the peak values regarding the compressive, tensile, and shear strengths of the specimens all decreased. Moreover, during the shear test with a certain number of dry–wet cycles, the larger the shear angle, the more serious impact of the dry–wet cycles on the shear behavior of the specimens was. These results indicate that the mechanical properties of nanocomposite fiber–CPB specimens are greatly affected by the alternation of dry and wet environments within a short time.
(2) With the increase in the curing age, the compressive strength and tensile strength of the specimens after the dry–wet cycling increased exponentially and finally stabilized after 28d of curing. The growth rate of peak stress gradually decreased during the whole process, indicating that with the increase in curing age, the mechanical properties of nanocomposite fiber–CPB are gradually reduced by the dry–wet cycling.
(3) The SEM results show that with the increase in the number of dry–wet cycles, the internal structure of the nanocomposite fiber–CPB material deteriorated gradually; the cracks and pores became more pronounced, leading to greater damage to the internal structural skeleton and an increased susceptibility to surface breakage and spalling.
(4) With the increase in curing age, the hydration products in the nanocomposite fiber–CPB materials became attached to the specimen surface, and the adhesion area gradually increased; at the same time, the number of cracks and holes on the surface decreased, and the failure process of the internal structure gradually decreased.

## Figures and Tables

**Table 1:** Performance parameters of glass fibers and nano-SiO_2_.

Material	Characterization	Parameters	Characterization	Parameters
Non-alkali glass fiber	Specification	6 mm	Fiber density	2.699 g/cm^2^
	Tensile strength	≥2000 Mpa	Elongation at break	≥2.5
	Tensile modulus of elasticity	≥85 GPa	Acid and alkali resistance	polar altitude
Nano-SiO_2_	Specific Surface Area	240 m^2^/g	Bulk density	0.06 g/cm^3^
	Density	2.2~2.6 g/cm^3^	Crystal type	Ball shape
	Color	White	Purity	≥99.99%

**Table 2:** Test equipment and equipment models.

Serial Number	Instrument/Equipment Name	Manufacturer
1	Electro-hydraulic servo press	Changchun Xinte Testing machine of China (Changchun, China)
2	SEM	Miero-source Detection (Hangzhou, China)
3	Jaw crusher	Xuzhou Sideli of China (Xuzhou, China)
4	Drying oven	Beijing Guangming Medical Equipment of China (Beijing, China)
5	Thermostatic water tank	Nanjing Xianou Manufacturing Company of China (Nanjing, China)
6	Standard curing box	Nanjing Jinrui Testing of China (Nanjing, China)

**Table 3:** The mass ratio of each component of CPB.

Material	Coal Gangune	Fly Ash	Cement	Glass Fiber	Nano-SiO_2_
Percentage	40%	50%	10%	0.3%	1%

**Table 4:** Test program.

Curing Age	Number of Wet and Dry Cycles	Mechanical Tests			Fine Structure Tests	Quantities
7 d	0, 1, 3, 7	Compressive	Tensile	Shear	SEM (10 mm × 10 mm × 5 mm)	60
		3	3	9	4	
14 d		Compressive	Tensile	Shear	SEM (10 mm × 10 mm × 5 mm)	60
		3	3	9	4	
28 d		Compressive	Tensile	Shear	SEM (10 mm × 10 mm × 5 mm)	60
		3	3	9	4	

**Table 5:** Shear strengths of the specimens at different curing ages.

Curing Age	Shear Strength/MPa		
	40°	50°	60°
7 d	0.97	0.75	0.32
14 d	1.07	0.71	0.47
28 d	1.47	1.12	0.48

## Data Availability

The data presented in this study are available on request from the corresponding author. The data are not publicly available due to privacy.

## References

[1] Amrani M., Taha Y., El Haloui Y., Benzaazoua M., Hakkou R. (2020). Sustainable Reuse of Coal Mine Waste: Experimental and Economic Assessments for Embankments and Pavement Layer Applications in Morocco. Minerals.

[2] Zhou N., Yao Y.N., Song W.J., He Z.W., Meng G.H., Liu Y. (2020). Present situation and prospect of coal gangue treatment technology. J. Min. Saf. Eng..

[3] Du J.L. (2021). Characteristics of coal gangue in Ximing Coal Mine and its significance of resource utilization. China Coal Geol..

[4] Gele J.R., Zhao R. (2021). Selection of unloading position for automatic waste discharging of truck transport based on EDEM. Coal. Eng..

[5] Fall M., Adrien D., Célestin J.C., Pokharel M., Touré M. (2009). Saturated hydraulic conductivity of cemented paste backfill. Miner. Eng..

[6] Fall M., Nasir O. (2010). Mechanical Behaviour of the Interface between Cemented Tailings Backfill and Retaining Structures under Shear Loads. Geotech. Geol. Eng..

[7] Simms P., Grabinsky M., Zhan G. (2007). Modelling evaporation of paste tailings from the Bulyanhulu mine. Can. Geotech. J..

[8] Ketov A., Rudakova L., Vaisman I., Ketov I., Haritonovs V., Sahmenko G. (2021). Recycling of rice husks ash for the preparation of resistant, lightweight and environment-friendly fired bricks. Constr. Build. Mater..

[9] Cui L., Fall M. (2016). Mechanical and thermal properties of cemented tailings materials at early ages: Influence of initial temperature, curing stress and drainage conditions. Constr. Build. Mater..

[10] Cui L., Fall M. (2017). Multiphysics Model for Consolidation Behavior of Cemented Paste Backfill. Int. J. Géoméch..

[11] He Y., Chen Q.S., Qi C.C., Zhang Q.L., Xiao C.C. (2019). Lithium slag and fly ash-based binder for cemented fine tailings backfill. J. Environ. Manag..

[12] Wang A., Cao S., Yilmaz E. (2022). Influence of types and contents of nano cellulose materials as reinforcement on stability performance of cementitious tailings backfill. Constr. Build. Mater..

[13] Behera S., Mishra D., Singh P., Mishra K., Mandal S.K., Ghosh C., Kumar R., Mandal P.K. (2021). Utilization of mill tailings, fly ash and slag as mine paste backfill material: Review and future perspective. Constr. Build. Mater..

[14] Ouffa N., Trauchessec R., Benzaazoua M., Lecomte A., Belem T. (2022). A methodological approach applied to elaborate alkali-activated binders for mine paste backfills. Cem. Concr. Compos..

[15] Aslani F., Dehghani A., Wang L. (2021). The effect of hollow glass microspheres, carbon nanofibers and activated carbon powder on mechanical and dry shrinkage performance of ultra-lightweight engineered cementitious composites. Constr. Build. Mater..

[16] Ghasabkolaei N., Choobbasti A.J., Roshan N., Ghasemi S.E. (2017). Geotechnical properties of the soils modified with nanomaterials: A comprehensive review. Arch. Civ. Mech. Eng..

[17] Jha K.K. (2012). An Energy Based Nanomechanical Properties Evaluation Method for Cementitious Materials. Ph.D. Thesis.

[18] Farzadnia N., Bahmani S.H., Asadi A., Hosseini S. (2018). Mechanical and microstructural properties of cement pastes with rice husk ash coated with carbon nanofibers using a natural polymer binder. Constr. Build. Mater..

[19] Fonseca C.S., Silva M.F., Mendes R.F., Hein P.R.G., Zangiacomo A.L., Savastano H., Tonoli G.H.D. (2019). Jute fibers and micro/nanofibrils as reinforcement in extruded fiber-cement composites. Constr. Build. Mater..

[20] Wang L., Lin X. (2020). Mechanical Properties and Micromechanism of Concrete Modified by Carbon Nanofibers. Concr. Cem. Prod..

[21] Qin R., Zhou A., Yu Z., Wang Q., Lau D. (2021). Role of carbon nanotube in reinforcing cementitious materials: An experimental and coarse-grained molecular dynamics study. Cem. Concr. Res..

[22] Gao B., Cao S., Yilmaz E. (2023). Effect of Content and Length of Polypropylene Fibers on Strength and Microstructure of Cementitious Tailings-Waste Rock Fill. Minerals.

[23] Song Y., Zhang L., Ren J., Chen J., Che Y., Yang H., Bi R. (2019). Study on damage characteristics of weak cementation sandstone under drying-wetting cycles based on nuclear magnetic resonance technique. J. Rock Mech. Eng..

[24] Hale P.A., Shakoor A. (2003). A Laboratory Investigation of the Effects of Cyclic Heating and Cooling, Wetting and Drying, and Freezing and Thawing on the Compressive Strength of Selected Sandstones. Environ. Eng. Geosci..

[25] Duda M., Renner J. (2013). The weakening effect of water on the brittle failure strength of sandstone. Geophys. J. Int..

[26] Yao H., Zhang Z., Zhu C., Shi Y., Li Y. (2010). Experimental study of mechanical properties of sandstone under cyclic drying and wetting. Rock Soil Mech..

[27] Wang Z., Liu X., Fu Y., Zhang L., Yuan W. (2016). Erosion analysis of argillaceous sandstone under dry-wet cycle in two pH conditions. Rock Soil Mech..

[28] Esfandiari Z., Ajdari M., Vahedifard F. (2021). Time-Dependent Deformation Characteristics of Unsaturated Sand–Bentonite Mixture under Drying–Wetting Cycles. J. Geotech. Geoenviron. Eng..

[29] Zhang F., Jiang A., Yang X. (2021). Shear creep experiments and modeling of granite under dry-wet cycling. Bull. Eng. Geol. Environ..

[30] Guo J.-J., Wang K., Guo T., Yang Z.-Y., Zhang P. (2019). Effect of Dry–Wet Ratio on Properties of Concrete Under Sulfate Attack. Materials.

[31] Huang K., Dai Z., Meng Y., Yu F., Yao J., Zhang W., Chi Z., Chen S. (2023). Mechanical behavior and fracture mechanism of red-bed mudstone under varied dry-wet cycling and prefabricated fracture planes with different loading angles. Theor. Appl. Fract. Mech..

[32] Cai X., Zhou Z., Tan L., Zang H., Song Z. (2020). Fracture behavior and damage mechanisms of sandstone subjected to wetting-drying cycles. Eng. Fract. Mech..

[33] Guo Y.B. (2022). Research on Properties of Coal-Based Solid Waste Nanocomposite Fiber Cementitious Materials.

[34] Cheng Q., Wang H., Guo Y., Du B., Yin Q., Zhang L., Yao Y., Zhou N. (2023). Experimental Study on Mechanical Properties of Coal-Based Solid Waste Nanocomposite Fiber Cementitious Backfill Material. Materials.

[35] Zhu C., Zhang J., Nan Z., Li M., He Z., Fu G. (2021). Effects of Doping Glass Fibers on the Early Strength of Sand-Based Cemented Paste Backfill for Solid Wastes. Adv. Civ. Eng..

[36] Ghafari E., Ghahari S., Feng Y., Severgnini F., Lu N. (2016). Effect of Zinc oxide and Al-Zinc oxide nanoparticles on the rheological properties of cement paste. Compos. Part B Eng..

[37] Liu X., Li Y., Wang W., Zhou Y., Cheng L., Fan Z. (2022). Research on mechanical properties and strength criterion of carbonaceous shale with pre-existing fissures under drying-wetting cycles. Chin. J. Rock Mech. Eng..

[38] Zhang A., Yang W., Ge Y., Du Y., Liu P. (2021). Effects of nano-SiO_2_ and nano-Al_2_O_3_ on mechanical and durability properties of cement-based materials: A comparative study. J. Build. Eng..

[39] (2016). Standard Test Method for Wetting and Drying Test of Solid Wastes.

[40] (2019). Standard for Test Methods of Concrete Physical and Mechanical Properties.

[41] (2009). Standard for Test Method of Performance on Building Mortar.

[42] Zhou Z., Cai X., Ma D., Chen L., Wang S., Tan L. (2018). Dynamic tensile properties of sandstone subjected to wetting and drying cycles. Constr. Build. Mater..

[43] Wang Z., Sun P., Zuo J., Liu C., Han Y., Zhang Z. (2021). Long-term properties and microstructure change of engineered cementitious composites subjected to high sulfate coal mine water in drying-wetting cycles. Mater. Des..

[44] Liu K., Gu T., Wang X., Wang J. (2022). Time-Dependence of the Mechanical Behavior of Loess after Dry-Wet Cycles. Appl. Sci..

